# Predicting Success of Allergen-Specific Immunotherapy

**DOI:** 10.3389/fimmu.2020.01826

**Published:** 2020-08-25

**Authors:** Ulrich M. Zissler, Carsten B. Schmidt-Weber

**Affiliations:** Center of Allergy and Environment (ZAUM), Technical University and Helmholtz Center Munich, Member of the German Center of Lung Research (DZL), and Member of the Helmholtz I&I Initiative, Munich, Germany

**Keywords:** allergen-specific immunotherapy, tolerance, biomarker, immune cells, epithelial cells, tissue homeostasis, rhinitis, asthma

## Abstract

The immune response to antigens is a key aspect of immunology, as it provides opportunities for therapeutic intervention. However, the induction of immunological tolerance is an evolving area that is still not sufficiently understood. Allergen immunotherapy (AIT) is a disease-modulating therapy available for immunoglobulin E (IgE)-mediated airway diseases such as allergic rhinitis or allergic asthma. This disease-modifying effect is not only antigen driven but also antigen specific. The specificity and also the long-lasting, often life-long symptom reduction make the therapy attractive for patients. Additionally, the chance to prevent the onset of asthma by treating allergic rhinitis with AIT is important. The mechanism and, in consequence, therapy guiding biomarker are still in its infancy. Recent studies demonstrated that the interaction of T, B, dendritic, and epithelial cells and macrophages are individually contributing to clinical tolerance and therefore underline the need for a system to monitor the progress and success of AIT. As clinical improvement is often accompanied by decreases in numbers of effector cells in the tissue, analyses of cellular responses and cytokine pattern provide a good insight into the mechanisms of AIT. The suppression of type-2 immunity is accompanied by decreased levels of type-2 mediators such as epithelial CCL-26 and interleukin (IL)-4, IL-13 produced by T cells that are constituting the immune memory and are increasingly controlled by regulatory T and B cells following AIT. Immune tolerance is also associated with increased production of type-1 mediators like interferon-gamma, tissue-homeostating factors like indoleamine 2,3-dioxygenase (IDO) expressed by macrophages and dendritic cells. Although these individual genes were convincingly demonstrated to play a role immune tolerance, they do not predict therapy outcomes of AIT on an individual level. Therefore, combinations or ratios of gene expression levels are a promising way to achieve predictive value and definition of helpful biomarker.

## Introduction

Allergen-specific immunotherapy (AIT) is practiced for more than 100 years now, but the understanding of the cellular and molecular mechanisms just evolved in the last 20 years. Good news is that several studies convincingly demonstrated clinical efficacy of injection as well as of sublingual treatment designs. While quite a few improvements in dosing, adjuvants, allergen extraction, allergen expression, and allergen modification were explored, the main treatment principle is the administration of the antigen into the host. In contrast to conventional vaccination against pathogens, the main target in allergen vaccination is to inhibit an already ongoing immune response. Thus, although the exact opposite reaction is intended, the applied intervention is the same: inject the antigen/allergen into the organism. In order to induce tolerance by the allergen vaccination, several adjustments have been used to obtain a tolerogenic effect such as the route of administration, extended vaccination schemes for several years, antigen doses, antigen modifications, tolerogenic adjuvants, and use of tolerogenic cell populations (1–5). Biomarker that monitor the antigen-specific induction of an immune response are available for conventional vaccination such as antigen-specific immunoglobulins (Ig's). In contrast, markers of tolerance induction are more difficult to identify, since the absence of inflammation and thus measurable markers are lacking. Ideal would be a marker that predicts whether a patient benefits from AIT before it evens starts. While the identification of such a marker is very difficult, research has taken smaller steps to approach this predictive biomarker that can be used to decide whether to apply ASIT or not. The smaller steps include marker that indicate whether allergen has been successfully administered (1.1), monitoring of anti-inflammatory effects (1.2), induction of immunosuppressive mechanisms (1.3), and prediction of treatment success following initiation of the treatment (1.4).

### Tracking Allergen Vaccination

Key diagnostic marker for any antigen-specific immune response is the Ig response and the conversion of initial IgM to high affinity maturated IgGs that demarcates the successful vaccination process. Exposure to environmental antigens as well as allergen-specific immunotherapy results in the induction of IgG4. This Ig isotype is distinguished from other Ig's by its constant region that it is not bound by complement factors and is bound only by low affinity to Fc receptors (6) and therefore not trigger proinflammatory responses. IgG_4_ is very efficiently induced even against those epitopes that were not recognized by patients' IgE (7). For this reason, IgG_4_ is currently the only generally accepted biomarker of AIT that demonstrates that the patient has received the therapeutic antigen (8). In context of allergy, it was demonstrated that IgG_4_ can block IgE binding sites and thereby mediates a welcome anti-inflammatory effect. This effect is particularly welcome in insect venom immunotherapy, where the prevention of IgE-mediated anaphylaxis is of particular value. Therefore, the potential of IgG_4_ in relation to IgE to bind to an allergen has been investigated intensively as a tolerance biomarker; however, its relationship to clinical symptoms is only visible in larger cohorts. Resolution of IgG_4_ responses to distinct epitopes revealed differential patterns in the recognition of allergens for insect venoms but did not show clinical outcomes (9). In addition, the ratio of IgG_4_ and IgE only correlates marginally to clinical symptoms (10). The inclusion of the IgG_4_ avidity demonstrated by serum-inhibition assays (facilitated antigen binding FAB) provides an improved view on the competition of IgE and IgG_4_. In this assay, it can be visualized that the ability to bind and compete with IgE is improving in AIT. A minor but significant correlation of facilitated allergen binding with combined daily symptom and medication scores was shown over a 3-week peak season at the first maintenance dose (week 8 of treatment); however, placebo samples were included in this analysis as well (11).

Due to differences in the components of the allergens, the picture can be very complex (12). In substitution therapies (e.g., hemophilia), the therapeutic recombinant protein (in this case factor VIII) is neutralized by antibodies of the IgG_4_ isotype (13) and destroys its catalytic activity. It is therefore the “bad guy” in this therapy as it inactivates the therapeutic agent. This example outside of AIT highlights the need for administration schemes that do not generate an immune response or at least promote tolerogenic recognition of the therapeutic antigen. Development in this direction could also be relevant for AIT and in particular for immunotherapies that use peptides rather than proteins. Peptide-specific immunotherapy does not induce IgG_4_, as it lacks three-dimensional epitopes for Ig binding and yet gives rise to a regulatory T cell response (14, 15). The clinical sustainability of peptide-induced tolerance could yet not be demonstrated convincingly, as high placebo signals prevented a successful conclusion of these trials.

Another interesting immunoglobulin isotype is IgA2, which is selectively transported through the epithelial surfaces. Allergen-specific IgA2 is increased by AIT, but in contrast to IgG_4_, these differences become only apparent 2 years after initiation of the treatment (16). IgA together with IgE and IgG_4_ is also present in salivary fluids and could be particularly interesting for sublingual immunotherapy (17).

### Detecting Decreasing Inflammation

Allergen-specific immunotherapy decreases local inflammatory, particularly type-2 cytokines such as IL-4, IL-5, or IL-13 following AIT; however. initial therapy even increases a broad spectrum of inflammatory responses including IL-36G, IL-8, CXCL-1, CXCL-2, and IL-1α, which was shown in nasal brushings (18). In contrast, interferon-gamma (IFN-γ) was found to be increased by AIT following 3 years of therapy (19). Clinical symptoms (FEV1, FVC, and FEF25–75%) correlate partially with bronchial lavage levels of IL-4 as well as IL-8 and most favorable with eosinophil counts (20). Several studies reported an initial increase in allergen-specific IgE. In particular, the initial increase in IL-4 may be counterproductive and act against tolerance induction. Inflammatory cytokines and transcription factors, specifically IL-4-induced GATA3, can bind and block the activation of the *FOXP3* promoter and thereby prevent the differentiation of AIT-induced regulatory T cells (Tregs) (21, 22). Cytokines induced in the vaccination phase can therefore be envisionaged as negative biomarker in AIT, which was also the basis for a clinical trial, where anti-IL-4 was used in the up-dosing phase to prevent antitolerogenic effects of IL-4 and thereby promote tolerogenic vaccination. In fact, the rise in allergen-specific Th2 cells in the up-dosing phase of AIT could be successfully reduced (5, 23). In addition to IL-4, also other inflammatory cytokines such as TNF family members, IL-1 or IFNs, may prevent tolerance induction (24–27). In order to limit these mediators as well, inhibitors could be imagined that block the activity and signal transduction of these mediators and thereby provide “tolerogenic adjuvants” for the vaccination. One idea is to use already clinically approved immune suppressors that temporarily block signal transduction of these cytokines. As these proinflammatory mediators, such as IL-4, often trigger the Janus kinase (JAK) pathway, the JAK inhibitor Tofacitinib represents one potential candidate for this approach. This was the background of an experimental immunotherapy where a JAK inhibitor was improving experimental tolerance induction, when it was used to cover the vaccination phase (1, 28). However, also other clinically used immunosuppressants such as glucocorticoid were suggested to support Treg cells at least in *in vitro* models (29), while cyclosporine A is counteracting the induction of these cells (30).

### Indicators of Immune Tolerance

Clinical unresponsiveness is often not identical with immunological tolerance; however, in other diseases, a relationship of induction of regulatory B cells (Bregs) in spontaneous clinical tolerance against kidney transplants was demonstrated (31). Bregs are able to suppress cells of the immune system by secretion of IL-10 (18, 32), IL-35 (33) and transforming growth factor beta (TGF-β) (32). IL-10^+^ B cells are a heterogenous group that can be separated to different subsets that demarcate distinct maturation phenotypes such as CD1d^hi^CD5^+^ (34, 35), CD24^hi^CD27^+^ (36), CD24^hi^CD38^hi^ B cell subsets (37), and CD25^+^CD71^+^CD73^−^ (38). The latter subset has been shown to be induced following AIT inhibiting antigen-specific CD4^+^ T cell proliferation and production of anti-inflammatory IgG_4_ antibodies. Induction of IL-10^+^ B cells is an early event in AIT, observed most abundantly within weeks of the up-dosing period (18). In the same study, FOXP3^+^ Tregs increased only after 3 years of therapy, and also the decrease in Th2 cells took as long thus much later as the induction of Bregs. At this time point, Bregs are back to baseline levels. In contrast, Th17 cells appeared also relatively early but are mainly considered as proinflammatory cells, despite the fact that they require the presence of the rather anti-inflammatory cytokine TGF-β (39, 40). Depending on certain circumstances and anatomic locations, T cells are described, which express FOXP3 and IL-17 at the same time and inversely correlated with Th2 cells (41). These cells or cells of a similar phenotype (FOXP3^+^IL-17^+^) also occur transiently in the first year of AIT (18). It may represent a transitory “Tr17” population, which possibly originates from Th17 cells and may further differentiate into fully regulatory T cells.

### Predicting Therapy Success: Hopeful or Helpful?

**Figure 1:**
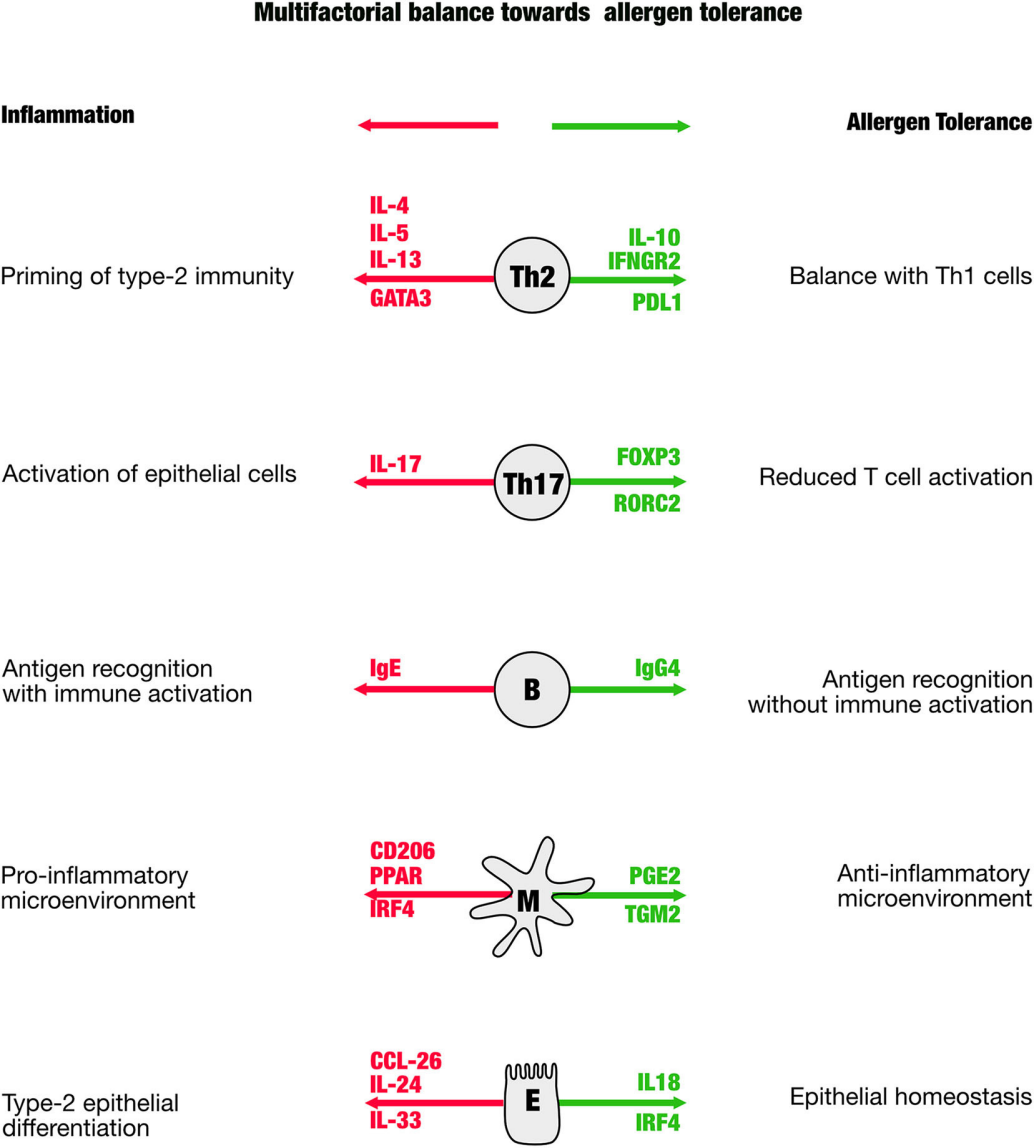
The scheme is illustrating distinct cellular mediators that have been shown to be involved in the immune regulation in allergen-specific immune tolerance. In green are shown genes that appear in the process of tolerance induction, in red are those that indicate a proallergic, inflammatory direction.

The prediction of therapy success, and in particular to support the physician to manage AIT to make it successful, is a major aim in biomarker research. The difficulty is that multiple proinflammatory players are in balance with those that act anti-inflammatory (Figure 1). As a consequence, a reliable biomarker will not be based on a single molecule, but rather need to cover multiple analytes that better represent the balance of the players contributing to tolerance. The first blood-borne biomarker that fulfills this idea is a ratio of Bregs and Th17 cells taken after the up-dosing phase, thus in the early phase of the therapy (18). This ratio correlates with the therapy success [*Retrospective Assessment of Seasonal Allergic Symptoms* (RAAS)] after 3 years and embraces the idea to include regulatory and inflammatory elements into account. The Bregs in the equation showed a high spread as well as the proinflammatory Th17 cells that were previously not considered in context of other AIT studies. Th17 cells are known to be very “plastic,” which means that they may not be fully differentiated and may have enough stemness to further differentiate even into regulatory T cells. This biomarker is awaiting to be validated in larger cohorts to be able to answer the question whether this marker is not only hopeful, but also helpful.

### Discussion and Outlook

The role of the tissue and tissue biomarker in the regulation of tolerance is still insufficiently investigated in AIT, while increasingly recognized as key factor in transplantation tolerance via mechanisms of amino acid consumption [auxotrophy; (42)]. The tissue interacts with the specific immune system either directly or indirectly via tissue-resident macrophages or dendritic cells. Differentiation of tissue-resident macrophages may provide important information and biomarker of how effective the allergen tolerance has been corrected by allergen-specific immunotherapy (43). Dendritic cells are key orchestrators of both the innate and the adaptive immune responses and are essential for the regulation of CD4^+^ T cell responses. When triggered by an allergen, immature DCs polarize into DC1s, DC2s, DC17s, or DCregs, which in turn can differentiate T cells into Th1 cells (DC1s), Th2 cells (DC2s), Th17 cells (DC17s), or regulatory T cells [DCregs; (44)]. Changes in expression of five combined DCreg/DC2-associated markers (CD141, C1Q, GATA3, FcγRIIIa, RIPK4) in peripheral blood mononuclear cells (PBMCs) correlated with clinical efficacy of sublingual immunotherapy (SLIT) at 2 and 4 months (44). The link between tissue cells, T cells, and macrophages or dendritic cells is regulated by CD8^+^ T cells, which recognize specific antigens on any cell via major histocompatibility complex class I (MHCI), while CD4^+^ T cells recognize allergen peptides only on MHCII expressed by dendritic cells. Consequently, CD8 cell may also play an important in direct tolerance induction (45), by interacting between tissue cells and antigen-presenting cells. Both CD4^+^ and CD8^+^ T cell cells produce interleukins that selectively act on tissue and epithelial cells that can mediate specific epithelial responses and can thereby contribute to clinical tolerance (46, 47). In turn, epithelial cells interact with all these cell types and innate lymphoid cells. Epithelial cells are directly responding to environmental influences and can therefore influence immune tolerance and immune homeostasis (48). Specifically, epithelial tissues respond to allergic inflammation or viral infections with distinct differentiation processes and commit toward E2 and E1 cells, respectively (49). These phenotypes can be detected in allergen-specific immunotherapy and are noninvasively detectable in secretions of the upper and lower airways (50). The important role of epithelial cells in tolerance regulation was also demonstrated in studies showing that airway epithelial cells can break immunotolerance upon recognition of bacteria and FcγRIII-mediated activation of the cells (51).

In conclusion, AIT induces clinical allergen tolerance that depends on multiple mechanisms across different immune and tissue cells. Therefore, an effective biomarker will consist of multiple analytes that cover different cellular processes. Whether multiple biomarkers as opposed to a single marker are helpful depends on simple devices that need to be developed and convert the hopeful experimental results into an helpful clinical routine.

## Author Contributions

UZ and CS-W wrote the manuscript and prepared the figure. All authors contributed to the article and approved the submitted version.

## Conflict of Interest

CS-W received research grants from Leti Pharma, Bencard, and Allergopharma. The remaining author declares that the research was conducted in the absence of any commercial or financial relationships that could be construed as a potential conflict of interest.
